# Supplementary material for: Holographic colour prints for enhanced optical security by combined phase and amplitude control
Source: Nat Commun. 2019 Jan 3;10:25. doi: 10.1038/s41467-018-07808-4 (PMC6318302; doi:10.1038/s41467-018-07808-4)
Supplement: Supplementary file 1 — Supplementary Information [file 41467_2018_7808_MOESM1_ESM.pdf]

Supplementary Information

Holographic Colour Prints for Enhanced Optical Security by Combined Phase and Amplitude Control

Lim *et al.*

Supplementary Methods

Supplementary Method 1 – Materials

Solvents were purchased from Sigma-Aldrich and used as-is. Photoresist (IP-dip, Nanoscribe GmbH) and glass substrates (fused silica, 25 mm squares with a thickness of 0.7 mm) were purchased from Nanoscribe GmbH.

Supplementary Method 2 – Sample fabrication

Direct laser writing was performed in a Photonic Professional GT system (Nanoscribe GmbH). A 780 nm femtosecond pulsed IR laser with a 90 fs pulse duration and 80 MHz repetition rate (Toptica FemtoFiber Pro) was focused into a puddle of liquid IP-dip photoresist by an immersion objective (Zeiss Plan Apo 63×, NA 1.4) to induce two-photon absorption and polymerisation. The lateral position of the laser spot was controlled by using galvanometric mirrors to deflect the beam within the field of view of the objective lens, whereas the axial position of the spot was controlled by using a piezoelectric and mechanical translation stage to shift the photoresist/substrate interface relative to the focal plane of the objective lens. This rastering of the laser spot created pixels and prints as cross-linked polymer structures on a glass substrate.

The laser power incident on the entrance aperture of the objective lens was controlled by an acousto-optic modulator (AA Opto-Electronic). For line exposures (blocks), the scan speed was 8000 μm s^{-1} and the laser power 21.0 mW for the first raster scan and 16.8 mW for the second raster scan. The hatching pitch was 250 nm and slice thickness 0.70 μm. For point exposures (pillars), the exposure time was varied between 0.02 and 0.04 ms and the laser power between 33.3 and 46.4 mW, and the slice thickness ranged from 0.69 to 1.01 μm. The slice thickness was adjusted to match the (dose-dependent) axial elongation of the point spread function of the laser spot while maintaining a vertical overlap of approximately 30% (300 to 430 nm depending on the size of the laser spot in the vertical direction).

To wash away the excess unexposed liquid photoresist, development was carried out by immersion of the sample in polyethylene glycol methyl ether acetate (PGMEA) for 5 minutes and then isopropyl alcohol (IPA) for 3 minutes, followed by transfer into nonafluorobutyl methyl ether (NFBME) as a low surface tension solvent for the final drying step. Due to the large difference in density between the two solvents, residual IPA carried over from the previous step would float on the surface of NFBME and had to be siphoned off before removing the sample. This step was necessary to minimise recontamination of the sample with IPA when it was withdrawn through the surface, as the IPA would otherwise dry on the sample and cause the pillars to collapse due to its relatively high surface tension.

Supplementary Method 3 – Phase plate thickness calibration

Using polymerised IP-dip photoresist with a refractive index of 1.54–1.58 across the visible spectrum, the block thickness required for 2π phase modulation is 0.79 μm, 0.95 μm, and 1.17 μm at our design wavelengths of 449, 527, and 638 nm. To span the required range of thicknesses and avoid unwanted shifts in filter colour at thicknesses below 0.6 μm, we used a thickness range of 0.6–1.8 μm. A series of blocks fabricated with different thicknesses in this range was scanned with a stylus profilometer (KLA Tencor) at a lateral speed of 10 μm s^{-1} and a force of 0.10 mg for thickness calibration. Based on the thickness errors we found, we estimated the limit of placement accuracy of the laser spot to be 100 nm in the axial direction. As such, we discretised the thickness in steps of no smaller than 100 nm, corresponding to quantised phase levels of 0.25π, 0.21π, and 0.17π for blue, green, and red light respectively. With a strict lower limit of 100 nm on the thickness step size, the number of phase levels used in the final prints was rounded down to 7, 9, and 11 respectively. Patterning too many phase levels is time-consuming and can be counterproductive as an unfavourable sequence of axial positioning errors from the piezoelectric stage could then cause undesirable reversals in the phase profile.

Supplementary Method 4 – Scanning electron microscopy

Scanning electron micrographs were acquired in a field emission scanning electron microscope (JEOL JSM-7600F) at an accelerating voltage of 5.0 kV and a working distance of 6.7 mm.

Supplementary Method 5 – Hologram computation

We ran our code in MATLAB R2017b on a Microsoft Surface Pro with an Intel i5-7300 2.60 GHz processor and 8 GB of RAM. Our design algorithm generated a 480×480 pixel three-colour multiplexed hologram in less than one minute.

Supplementary Method 6 – Simulations

The holographic projections in Supplementary Figs. 10 and 11 were simulated based on the output of our design algorithm (a phase map and an amplitude map) using the same source images as in Figs. 1, 3, and 4. The simulated projections were calculated from the phase and amplitude maps in MATLAB as follows. First, the element-wise product of the phase map (in phasor form) and the amplitude map is computed, approximating the initial electric field distribution of light immediately after being transmitted through the print. Then a Fourier transform is taken, approximating the propagation of light into the far field (in the Fraunhofer limit). Lastly, the square modulus is taken to convert the electric field strength into an intensity image, shown in logarithmic scale to approximate the human visual response.

Supplementary Method 7 – Photography of holographic projections

Holograms were projected in transmission onto a white wall and photographed using a DSLR camera in a darkened room. Coherent illumination was provided by 638 nm red, 527 nm green, and 449 nm blue laser diode modules with a maximum power of 4.5 mW (ThorLabs). Within our setup, we measured the actual power at the sample to be approximately 2 mW on average. The distance of the holographic colour prints from the wall (projection distance) was 135 cm, at which the holographic projections measured between 10 and 15 cm across. Photographs of the holographic projections in Figs. 3 and 4 show a ~20 cm square region centred on the projection.

Supplementary Method 8 – Optical characterisation

Optical micrographs and spectra were acquired in a Nikon Eclipse LV100ND optical microscope equipped with a CRAIC 508 PV microspectrophotometer and a Nikon DS-Ri2 camera. Samples were backlit by halogen lamp illumination and measured in transmission through a 5×/0.15 NA objective lens. As the colour filters are diffractive in nature, wavelengths that pass are transmitted on-axis while rejected wavelengths are diverted off-axis. Thus we measure the transmittance spectra in a narrow cone of acceptance angles using an objective with a numerical aperture of 0.15 (a half-angle of 8.6°).

Supplementary Method 9 – Colour filter selection

Spectra were analysed by comparing their average transmittance within three narrow wavelength bands centred at the red, green, and blue laser wavelengths, $\bar{T}_R$, $\bar{T}_G$, and $\bar{T}_B$. For each spectrum, these values were used to calculate figures of merit χ_R, χ_G, and χ_B that determine the suitability of the corresponding pillar array for use as a red, green, or blue colour filter. For example, the figure of merit for a red colour filter χ_R is the sum of the difference between red and green transmittances and the difference between red and blue transmittances, i.e. $\chi_R = (\bar{T}_R - \bar{T}_G) + (\bar{T}_R - \bar{T}_B) = 2\bar{T}_R - \bar{T}_G - \bar{T}_B$. A matrix representation was used in the code to enable vectorisation of the actual calculations.

Although a more accurate figure of merit should take into account the wavelength selectivity for each possible set of filters as a group (as described in the next section) rather than for individual filters, we did not adopt this in our design algorithm as it would greatly increase the number of computations.

Supplementary Notes

Supplementary Note 1 – Colour palette and wavelength selectivity

The range of colours attainable by varying the pillar dimensions (height and diameter) is shown in Supplementary Fig. 1. Pillars on blocks (Supplementary Fig. 1b,c) give darker colours that are spectrally shifted from the colours of pillars patterned directly on the glass substrate (Supplementary Fig. 1a). As such, we chose the block thicknesses for the prints to lie in a range (0.6–1.8 μm) over which the pillars showed little to no colour change with block thickness (Supplementary Fig. 1c), so as to afford relatively thickness-independent colour filters. We then averaged the spectra measured for colour filters with blocks of thickness in this range to minimise the effects of any remaining thickness dependence on designing prints. The similarity between the colour space plots in Supplementary Fig. 2a (averaged) and Supplementary Fig. 2b (1.0 μm thick) shows that there was in fact almost no thickness dependence remaining even before averaging. The coverage relative to sRGB of the averaged spectra was 53%, wide enough for us to pick out suitable filters for colour prints. Supplementary Table 1 lists the dimensions of pillars and the thickness-averaged RGB transmittances of the colour filters in the *Perfume* print.

By performing experiments to explore the parameter space of pillar dimensions, we were able to identify a set of three colour filters with adequate wavelength selectivity for multiplexing RGB holograms. We define the wavelength selectivity of transmission as the ratio of transmittances at the design wavelength for a colour filter designed to pass it and a colour filter designed to reject it. The RGB wavelength selectivity for the chosen set of colour filters is calculated in Supplementary Table 2, and ranges from 3.2 to 6.6 among the red, green, and blue colour filters, for an average selectivity of 4.6.

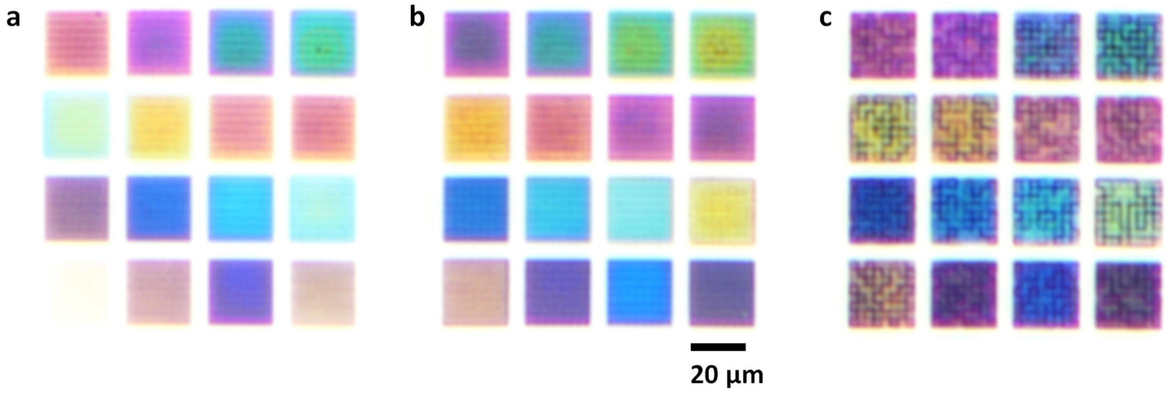

***Supplementary Figure 1: Colour palettes derived from pillar array colour filters by varying the pillar dimensions of height and diameter*. (a)** *Without blocks underneath (i.e. with a block thickness of 0 µm),* **(b)** *with blocks of thickness 1.0 µm under the pillars, and* **(c)** *with blocks of random thicknesses in the range 0.6–1.8 µm under the pillars. The pitch of the pillar arrays is 1.0 µm in all cases.*

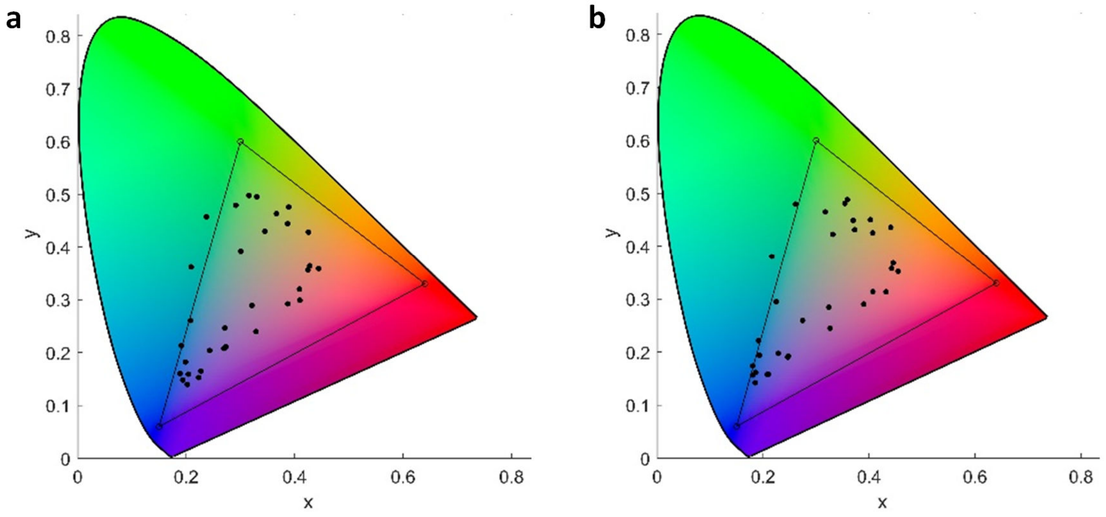

***Supplementary Figure 2: Comparison of filter colours under different conditions of block thickness.* (a)** *Colour space plot for colour filters on blocks of thicknesses 0.6, 1.0, 1.4 and 1.8 µm, with colours converted from thickness-averaged spectra for a 1931 CIE 2° standard observer viewing under illuminant D65 (white light with a colour temperature of 6500 K). For these colour filters, the pillar height and diameter were varied in the ranges 0.5–2.7 µm and 310–390 nm respectively, achieving a 53% coverage of the sRGB colour gamut.* **(b)** *Colour space plot for colour filters on blocks of thickness 1.0 µm with no averaging performed. There are no major differences between plots (a) and (b) as the colour is almost independent of block thickness in this thickness range (0.6–1.8 µm).*

Supplementary Table 1: Dimensions of pillars and RGB transmittance values for the colour filters in the* Perfume *print. *The transmittance spectra were averaged over blocks of thicknesses 0.6, 1.0, 1.4 and 1.8 μm, and the transmittance values further averaged over a narrow bandwidth of 4 nm centred at the wavelengths 449 nm (blue), 527 nm (green), and 638 nm (red), as well as a broadband spectral range of 450–650 nm (white).*

**Colour**	**Nominal Height**	**Nominal Diameter**	**Transmittance**			
			Blue (449±2 nm)	*Green (527±2 nm)*	*Red (638±2 nm)*	*White (450–650 nm)*
Red	1.9 μm	390 nm	21.0%	9.8%	53.7%	24.9%
Orange	1.7 μm	380 nm	23.2%	24.0%	71.2%	39.8%
Yellow	1.5 μm	380 nm	13.7%	42.3%	68.3%	47.7%
Green	2.6 μm	390 nm	14.1%	64.8%	9.2%	36.5%
Blue	0.7 μm	380 nm	67.8%	18.1%	15.5%	22.3%
Purple	1.1 μm	300 nm	59.7%	13.6%	22.6%	21.0%

Supplementary Table 2: RGB wavelength selectivity of the colour filters in the* Perfume *print*. The selectivity values are calculated from the transmittance values in Supplementary Table 1. Mutual selectivities among the red, green, and blue colour filters are highlighted in bold while those involving the yellow filters (not used for multiplexing) are greyed out.*

Selectivity of transmission	at blue wavelength	at green wavelength	at red wavelength
purple filter vs green filter	59.7% ÷ 14.1% = 4.2		
purple filter vs yellow filter	59.7% ÷ 13.7% = 4.4		
purple filter vs orange filter	59.7% ÷ 23.2% = 2.6		
purple filter vs red filter	59.7% ÷ 21.0% = 2.8		
**blue filter vs green filter**	67.8% ÷ 14.1% = **4.8**		
blue filter vs yellow filter	67.8% ÷ 13.7% = 4.9		
blue filter vs orange filter	67.8% ÷ 23.2% = 2.9		
**blue filter vs red filter**	67.8% ÷ 21.0% = **3.2**		
green filter vs purple filter		64.8% ÷ 13.6% = 4.8	
**green filter vs blue filter**		64.8% ÷ 18.1% = **3.6**	
green filter vs yellow filter		64.8% ÷ 42.3% = 1.5	
green filter vs orange filter		64.8% ÷ 24.0% = 2.7	
**green filter vs red filter**		64.8% ÷ 9.8% = **6.6**	
orange filter vs purple filter			71.2% ÷ 22.6% = 3.2
orange filter vs blue filter			71.2% ÷ 15.5% = 4.6
orange filter vs green filter			71.2% ÷ 9.2% = 7.7
orange filter vs yellow filter			71.2% ÷ 68.3% = 1.0
red filter vs purple filter			53.7% ÷ 22.6% = 2.4
**red filter vs blue filter**			53.7% ÷ 15.5% = **3.5**
**red filter vs green filter**			53.7% ÷ 9.2% = **5.8**
red filter vs yellow filter			53.7% ÷ 68.3% = 0.8
yellow filter vs purple filter		42.3% ÷ 13.6% = 3.1	68.3% ÷ 22.6% = 3.0
yellow filter vs blue filter		42.3% ÷ 18.1% = 2.3	68.3% ÷ 15.5% = 4.4
yellow filter vs green filter		42.3% ÷ 64.8% = 0.7	68.3% ÷ 9.2% = 7.4
yellow filter vs orange filter		42.3% ÷ 24.0% = 1.8	68.3% ÷ 71.2% = 1.0
yellow filter vs red filter		42.3% ÷ 9.8% = 4.3	68.3% ÷ 53.7% = 1.3

Supplementary Note 2 – Balancing wavelength selectivity in multiplexed holograms

In a multiplexed hologram, the overall transmission efficiency for a given channel is the product of the area fraction occupied by the channel and the weighted average of the transmittance of the colour filters on that channel, with an upper bound of 33% for the case of equal area fractions in an RGB hologram. If unequal area allocation arises from a predominance of one or two colours in the colour image to be printed, this can be compensated by adjusting the colour balance of the image before colour matching. Alternatively, it can also be desirable to deliberately encourage an unequal area allocation when the wavelength selectivity of the filters on one channel is significantly worse than those on others. In this manner, the number of the total hologram pixels allocated to each channel can be adjusted to balance out the transmission characteristics of the filters. For example, if the desired green transmission of the green filters (signal) does not sufficiently exceed the unwanted green transmission of the red and blue filters (noise, which manifests as crosstalk), more green pixels can be allocated to increase the signal-to-noise ratio on the green channel.

Here, a useful metric is the signal strength, which we define for each channel as the product of its area fraction and its average transmittance at its design wavelength. Noise strength terms can be defined analogously as the product of the area fraction of a channel and its average transmittance at the design wavelengths of other channels. We construct a matrix with the signal strengths on the diagonal and the noise strengths as off-diagonal (cross) terms, where the signal-to-noise contrast across all channels is balanced when it most closely approximates a diagonal matrix with a constant baseline shift.

Using this signal-to-noise matrix, we found that the appearance of crosstalk in the holographic projections was minimised by applying a slight green tint to the colour balance of the source image for the *Perfume* print in Fig. 4 to give a pixel allocation of 36% in the green channel, 29% in the red channel, 27% in the blue channel, and 8% of yellow pixels not assigned to any channel. This green tint is not obvious in the final printed image, but is only used in the design stage to promote a desired colour matching outcome.

Supplementary Note 3 – Practical applicability of holographic colour prints

For practical application of our holographic colour prints as optical security devices, the prints should be usable under non-ideal conditions and without the aid of a specialised viewing setup. Thus, the colour images should not require a microscope to be seen, and the holographic projections should be robust to deviations in the illumination angle and easily viewed with a standard laser pointer even in the presence of ambient background light.

Supplementary Fig. 3a demonstrates the possibility of viewing our holographic colour prints using a handheld phone camera with a low cost macro lens attachment. The QR code colour image can be clearly seen although fine details cannot be resolved as the macro lens we used did not have a high enough magnification. We note that while we used illumination from a microscope condenser here for convenience, we are also able to use a focusable or collimated flashlight to illuminate our colour prints as long as it produces a sufficiently narrow beam. A narrow beam is required because of the diffractive nature of the colour filters, as explained in the following.

The colour filters separate incident light into two components: one that is transmitted on-axis (the desired colour) and another that is diffracted away from the optical axis (the complementary colour, which is unwanted). For the intended subtractive colour effect to be produced, we must collect only the desired colour. If the collection angle of the lens is too large, both components are collected and the colour becomes washed out as they recombine to give the colour of the light source. Alternatively, if illumination is delivered over too wide a range of angles, the angular separation between the two components is lost and again both are collected. Thus the range of illumination and collection angles, i.e. the combined numerical aperture of the imaging system, must be considered when attempting to view our colour prints. Since the collection numerical aperture is set by the macro lens, we instead control the illumination numerical aperture. Based on the numerical aperture dependence observed in Supplementary Fig. 3b, the diffractive colour can still be seen up to a numerical aperture of 0.4, or a beam angle of up to ±23°, which is achievable by many common commercially available flashlights.

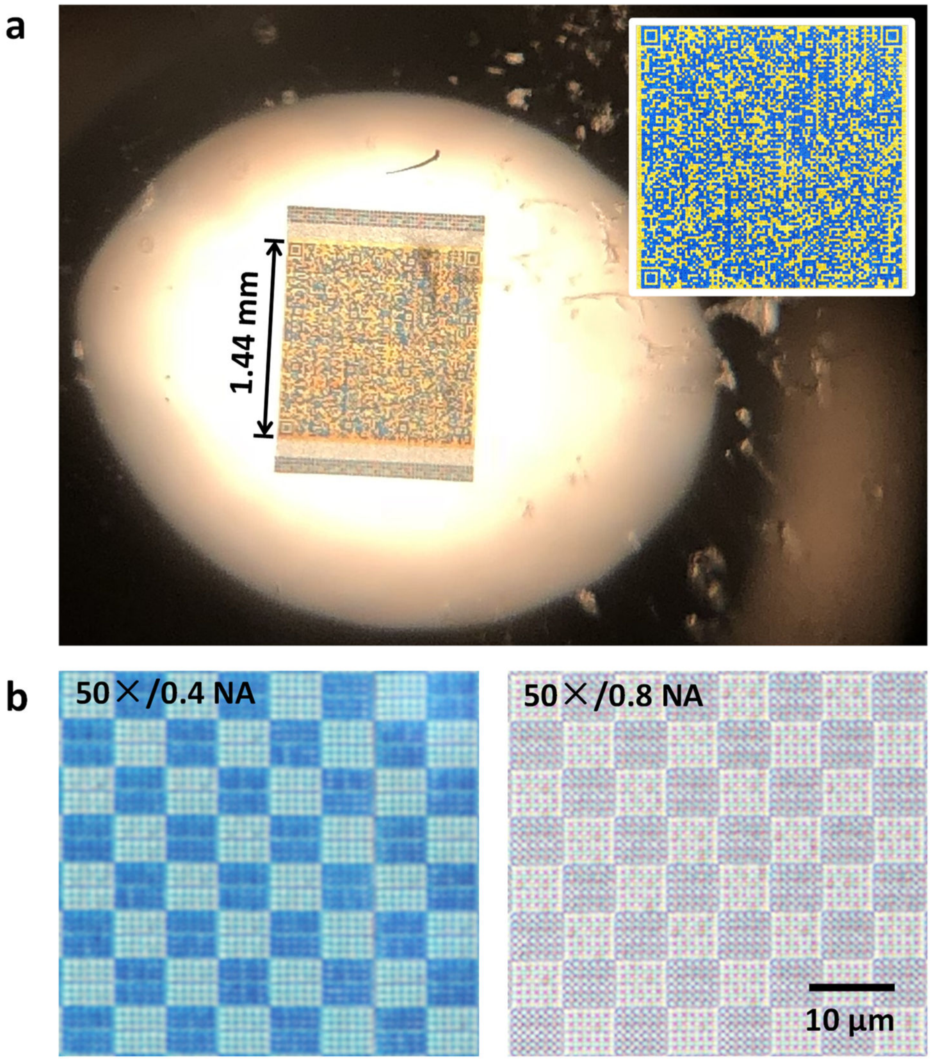

Supplementary Figure 3: Structural colour images viewed under different numerical aperture conditions. (a) *Photograph of the QR code print taken using a handheld phone camera (Apple iPhone 8 Plus) mounted with a low cost 10× macro lens attachment (Shuohu). The sample was backlit with white light transmission illumination from a microscope condenser set to a small numerical aperture of 0.1 for optimal viewing. Any white light source with a narrow beam angle could also be used for illumination. The inset shows the optical micrograph from Fig. 3c for comparison.* ***(b)*** *Comparison of pillar array colour filters imaged with a 50×/0.40 NA microscope objective (left) and a 50×/0.80 NA microscope objective (right) with the condenser set to a matching illumination numerical aperture. The illumination numerical aperture must be limited to below 0.4 so that the large collection numerical aperture of a macro lens or high magnification objective does not wash out the diffractive colour.*

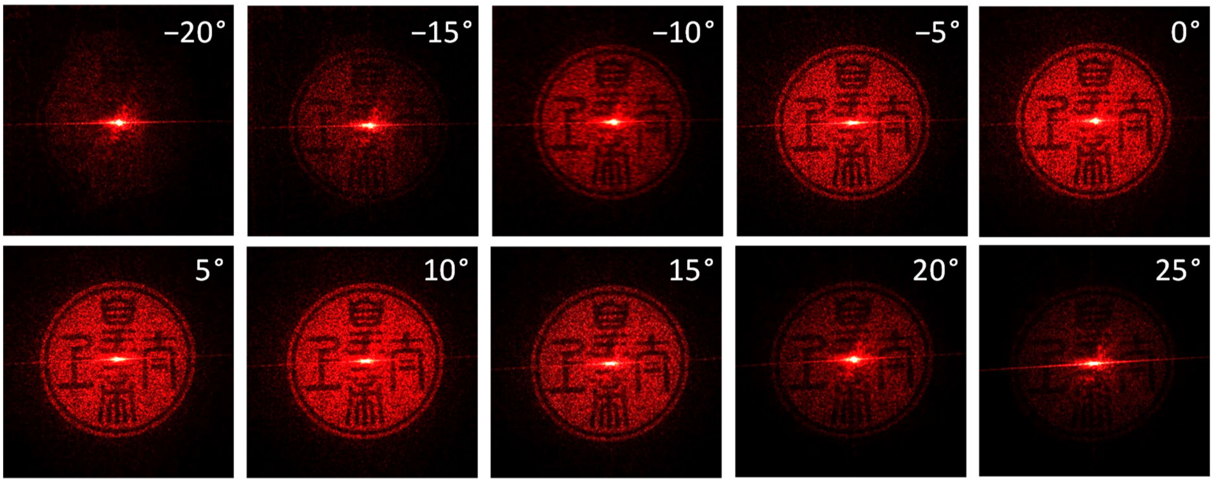

Supplementary Figure 4: Chinese seal holographic projection viewed at various illumination angles. *Illumination angles are specified in the top-right corner of each image, with 0° being normal incidence. Positive (negative) angles represent clockwise (anticlockwise) rotations of the print, where the print was rotated to the right (left) while the laser and screen were not adjusted. The projection is maintained over a ~30° range of illumination angles, only fading away at −15° to −10° on the left and 15° to 20° on the right.*

To investigate the angle dependence of our prints under laser illumination, we varied the angle at which the beam was incident on a print and photographed the holographic projections as before. Supplementary Fig. 4 shows the result of this test on the Chinese seal projection of the QR code print. The projection is essentially angle-insensitive between −5° and 10°, and suffers a slight decrease in brightness at −10° and 15° with little loss in quality. The projection is faintly visible at −15° and 20° and disappears as the angle is increased farther. (The slight asymmetry in the usable range of illumination angles might be due to a small average tilt in the pillars of 2–3° relative to the normal, possibly introduced during the drying step of the development process.) Due to the ~30° range of illumination angle tolerance, it is easy to project the holograms by holding the print in one hand and a laser pointer in the other.

Supplementary Fig. 5 illustrates the wide-angle characteristics of the print, comparing the appearance of the projection at different distances from the print to the screen. At a distance of 20 cm, the projection size of 2 cm is too small to avoid significant contamination with the central undiffracted zero-order spot. Other undesirable features are also present: the projection is repeated at higher diffraction orders away from the centre, and at even higher angles, the projection from the other channel and its higher diffraction orders can be seen. (As explained in the figure caption, because the colour filters work by diffracting unwanted wavelengths off-axis, the unwanted projections still appear, but only at very high angles.) Fortunately, these problems are easily solved by simply using a longer projection distance on the order of 1 m or farther. At a distance of 135 cm, the projection has expanded to a size of 12.5 cm and its features can be more clearly discerned even in the presence of the zero-order spot. The higher diffraction orders (which contain much less power) are weak enough that they can barely be seen, and the unwanted projections from the other channel are far off to the sides, over 70 cm away. We thus use a projection distance of 135 cm and show photographs of only a ~20 cm square containing the central projection in Figs. 3 and 4.

We note that the relative angle independence of the projections and the repeating of the projections at higher orders are both characteristic of holograms in the so-called “thin hologram” regime, which applies to our holograms as their thickness of 0.6–1.8 µm is smaller than their pixel pitch of 3 µm. (This is unlike the case of “thick” volume Bragg gratings for which the thickness is much larger than the pitch, which results in sharply angle-dependent projections in only the zero diffraction order.)

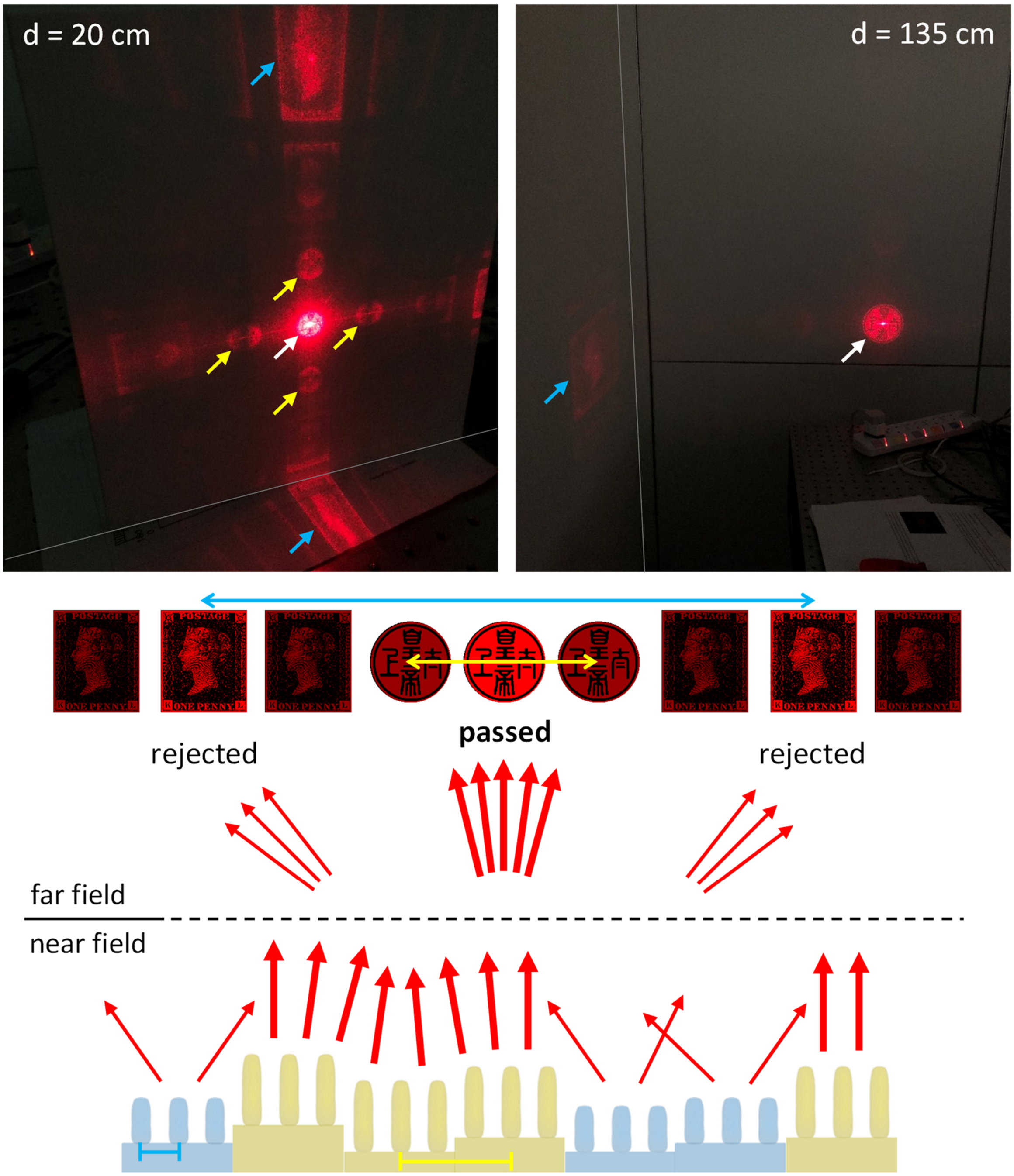

Supplementary Figure 5: Wide-angle characteristics of the Chinese seal holographic projection. *Photographs are taken at different projection distances* d*, with the sample placed: (left) near a white sheet of paper* (d = 20 cm) *and (right) far from a white wall* (d = 135 cm). *The central projection (white arrow) is surrounded by higher diffraction orders of the projection (yellow arrows). At higher angles, the Penny Black stamp holographic projection from the other channel (blue arrows) can be seen. The undesirable features that disturb the central projection at* d = 20 cm *are much less apparent at* d = 135 cm, *at which the projection has expanded from 2 cm to 12.5 cm, the higher orders have faded, and the*

unwanted projection is now over 70 cm away from the main projection. Thin grey lines mark boundaries where the screen makes a right angle (between two sheets of paper on left, and between two walls on right). The schematic (bottom) illustrates the workings of the colour filters and holograms: red light is passed (thick red arrows) by the yellow colour filters and rejected (thin red arrows) by the blue colour filters, creating a diffraction pattern in the far field upon which the holographic projections are superimposed. Because the diffraction angle is inversely related to the pitch, the hologram phase plates (pixel pitch 3 μm, yellow scale bar) only weakly modulate the angles of the incident light as compared to the colour filters (pillar pitch 1 μm, blue scale bar), which divert the light far off-axis when they reject it. As a result, the unwanted projections are well separated from the main projection at 3× the angular separation from the centre (blue double-headed arrow) as compared to the first-order peaks (yellow double-headed arrow). Note that the projections photographed at d = 20 cm *and* d = 135 cm *are both well within the far field.*

We note that although we have photographed the projections in a darkened room for clarity, this is not a requirement for viewing the holograms. The laser power of 2 mW that we used afforded bright projections that could be clearly seen under standard room lighting conditions (Supplementary Fig. 6). Similar results were obtained using common commercially available Class 3R laser pointers with an output power of up to 5 mW. The large size of the projections also makes for convenient viewing of the holograms.

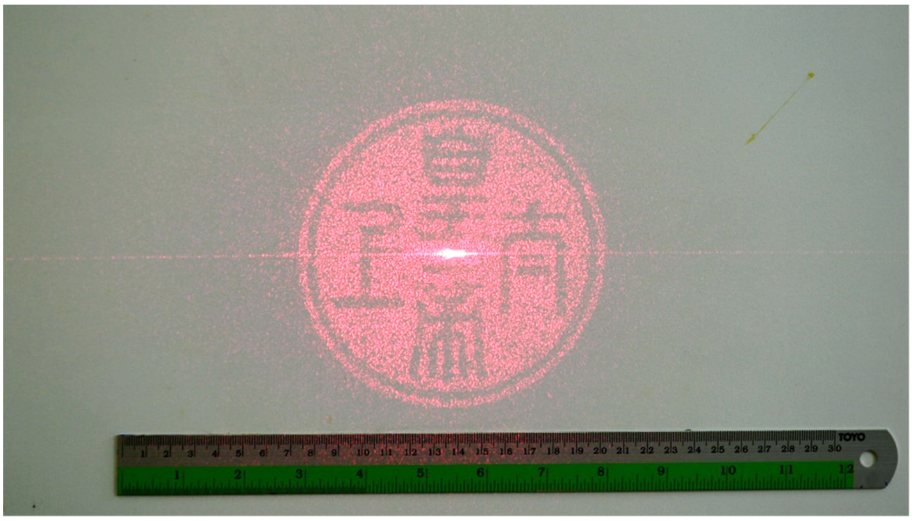

Supplementary Figure 6: Ease of viewing the Chinese seal holographic projection under ambient lighting. *The projection is bright and clear in the presence of strong background light in the room despite using only 2 mW of laser power. Projected on a white wall 135 cm away, the projection measures 12.5 cm across.*

Because of the on-axis nature of the projections, the projected images overlap perfectly as long as their illumination sources are collimated and collinear (beams sharing the same axis). As seen in Supplementary Fig. 7, colour mixing of the red and blue lasers to give purple occurs in the overlap of the projections. This demonstrates the potential of our method to achieve multi-colour projection and show full colour holograms.

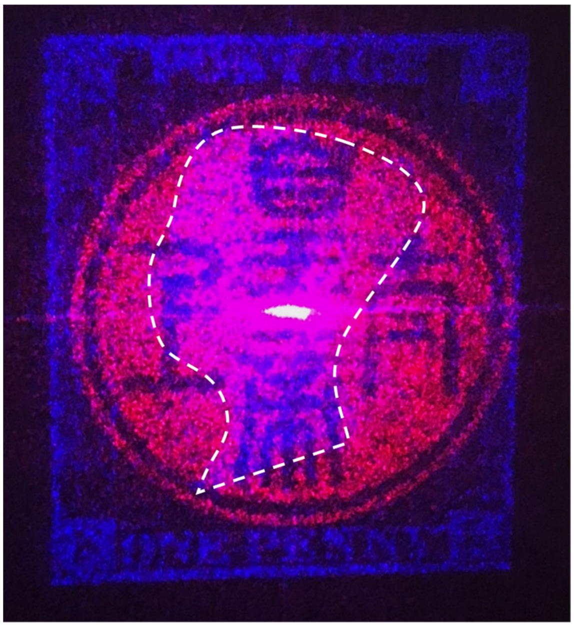

Supplementary Figure 7: Simultaneous two-colour holographic projection from the QR code print. *Under illumination with collinear red and blue lasers, the side profile of Queen Victoria (white dashed outline) in the centre of the Penny Black stamp (blue) overlaps with the Chinese seal (red) and so appears magenta, demonstrating the possibility of achieving multi-colour projection. The projection distance is 135 cm.*

Supplementary Note 4 – Measuring amplitude-phase coupling

As described in the main body of the paper, phase-amplitude coupling was minimised using a range of phase plate thicknesses that produced only minor variations in the colour of the pillar colour filters. Hence, we have experimentally shown that phase variation would not greatly affect the amplitude. However, amplitude-phase coupling might still be present, i.e. the pillar colour filters might contribute an additional phase shift on top of that imparted by the underlying phase plates, such that control of amplitude also affects the phase. If this unwanted additional phase shift is significant and uncompensated, it could disrupt the holographic projections in multi-colour prints such as the *Perfume* print, which have more than one colour in each colour channel. (Amplitude-phase coupling would not affect the QR code print because it imposes a uniform phase shift on each colour channel, which has no effect on the projections.)

To quantify any phase shift caused by the pillar colour filters, we fabricated and compared binary phase gratings with phase elements consisting of either: (1) phase plate blocks of two different thicknesses, or (2) two sets of pillars with different dimensions arrayed on top of a base layer of blocks of uniform thickness. The phase plate grating (1) uses blocks of 1.0 and 1.5 μm thickness, which were chosen to produce relative phase shifts of approximately 0 and π across the visible spectrum. For the pillar array gratings (2), we chose pillars with significantly different dimensions to maximise their phase difference, but with similar transmittances at the design wavelength so as to achieve a relatively flat amplitude profile across the grating. Under these conditions, any diffraction observed would be due primarily to a periodic phase variation created by the phase difference between the two sets of pillar arrays. Then comparing the power in the diffraction orders of gratings (1) and (2) allows us to directly compare the strength of phase modulation by blocks and pillar arrays. Supplementary Fig. 8 shows the fabricated gratings and their dimensions.

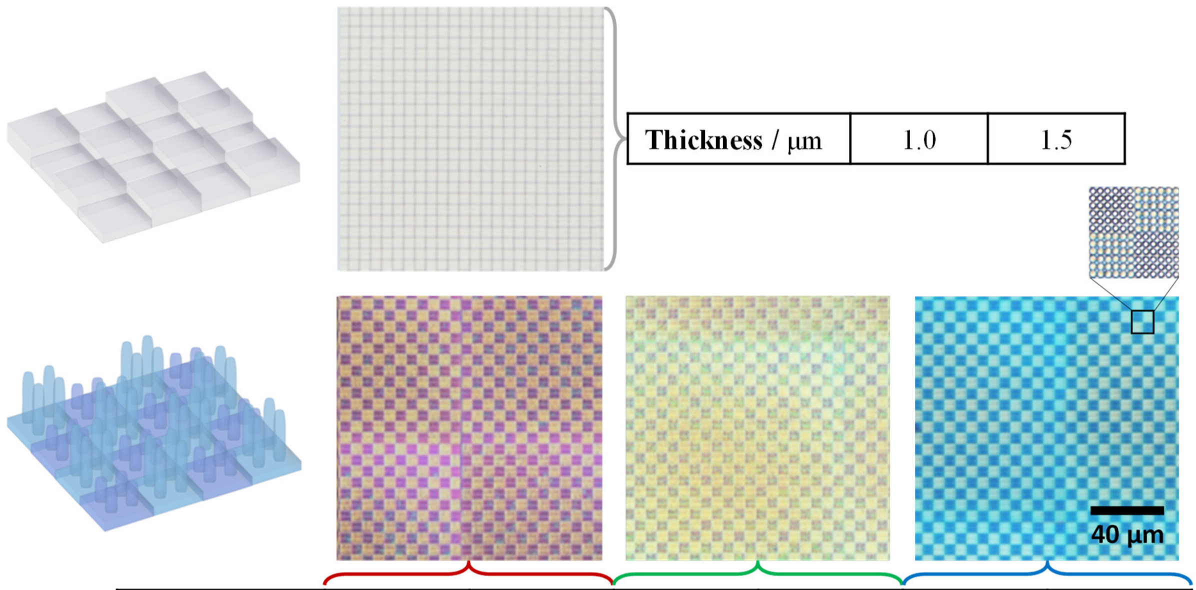

Thickness / µm	1.0	1.5

Height / µm	1.9	1.4	2.6	1.3	0.7	1.1
Diameter / nm	390	380	390	380	380	380

Supplementary Figure 8: Binary phase gratings made of phase plates and pillars. *Transmission optical micrographs of checkerboard binary phase gratings composed of: (top) phase plates of thickness 1.0 and 1.5 µm, and (bottom, from left) red, green, and blue pillars respectively, imaged with a 10×/0.20 NA objective. The inset was imaged with a 100×/0.90 NA objective. Schematics of the two grating types are shown on far left. The full checkerboards have 240×240 squares, where each square is a 2×2 super-pixel of holographic colour pixels (3×3 pillar array on top of a 3×3 µm² block) as shown in the inset. In the pillar array gratings, the blocks form a constant 1.0 µm thick base layer while the squares alternate between two slightly different colours (different pillar dimensions) that have a similar transmittance at the design wavelength.*

We observed that the power diffracted into the first order by the phase plate grating was more than ten times of that diffracted by the pillar array phase grating (Supplementary Fig. 9). This result shows that the colour filters do affect the phase of the transmitted light, but their effect is an order of magnitude smaller than that of the phase plate blocks. As such, it is reasonable to neglect amplitude-phase coupling in the design of holographic colour prints as we have done here. We note that full characterisation of the phase imparted would allow straightforward correction of this coupling in the design stage by changing the underlying block thickness in each pixel to compensate for the extra phase, which could potentially improve the quality of the projections by a factor of ~10%.

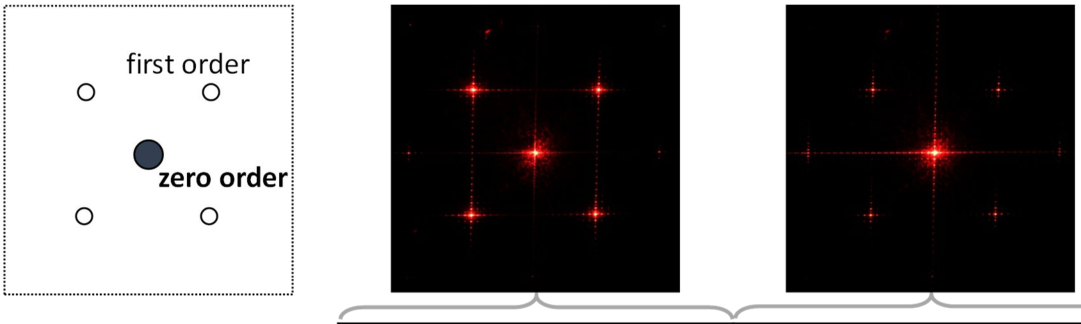

		**Phase plate grating**			**Pillar gratings**		
	**Laser colour**	Red	Green	Blue	Red	Green	Blue
**Power** / mW	**Zero order**	0.970	1.020	0.688	1.100	1.030	0.747
	**First order**	0.601	0.585	0.546	0.044	0.032	0.053

Supplementary Figure 9: Comparison of the diffracted power for phase plate gratings and pillar array gratings. *As labelled in the top-left schematic, the power in the zero order is directly measured from the central bright spot (filled circle) while the power in the first order is summed over the four diffraction peaks (open circles) produced by the checkerboard gratings. The photographs of the diffraction patterns under red laser illumination show zero order spots of similar intensity but much stronger first order peaks for the phase plate grating, as confirmed by the measurements in the table. Misalignment errors during stitching of the write-fields in these prints introduce an additional periodicity along the x- and y- directions, which superimposes a horizontal and vertical flare on all the diffraction peaks. The flare does not substantially alter the results of the experiment as it reduces the power in each peak by an equal percentage. The same phase plate grating is measured with all three lasers (638 nm red, 527 nm green, and 449 nm blue) while each pillar array grating is measured with the laser of the corresponding colour.*

Supplementary Note 5 – Efficiency measurements

We calculated the transmission and diffraction efficiency of the QR code print in Supplementary Table 3. For simplicity, we ignore the diffractive nature of the pillar colour filters and treat them as transmissive elements – the unwanted projections diffracted off-axis are considered to be rejected, while the on-axis central projection is passed (Supplementary Fig. 5). In this manner, we define the filter transmission efficiency on each channel as the ratio of the power in the central projection (transmitted through the print) to the power transmitted through bare glass at the design wavelength. Having accounted for the transmission characteristics, we then define the hologram diffraction efficiency as the ratio of diffracted power (power in the central projection but not in the zero-order spot) to the power in the central projection.

The filter transmission efficiency of the QR code sample was measured using a power meter placed immediately before and after the sample. The transmission efficiency was measured to be 32% for blue and 34% for red laser illumination, close to the expected value of 34% for a ~50% area fraction of pixels with a transmittance of 68% (blue pixels at blue laser wavelength and yellow pixels at red laser wavelength). The hologram diffraction efficiency is 72% for the blue projection and 47% for the red projection. The overall efficiency of the print, calculated as the product of the transmission efficiency of the glass substrate, the filter transmission efficiency, and the hologram diffraction efficiency, is 21% for blue and 14% for red. These efficiencies are sufficient for the holographic projections from our prints to be visible at low laser power and in a bright environment as seen in Supplementary Fig. 6. Further improvements in the hologram efficiency and overall efficiency could be made if future advances in the axial positioning accuracy of the laser writer allow the phase plate thickness profile of the holograms to more closely approximate their ideal phase profiles (Supplementary Method 3).

We also calculated the on-axis transmission efficiency of the *Perfume* print in Fig. 4 for showing a colour image under white light illumination. Taking the weighted average of the filter transmittances for white light (Supplementary Table 1) with the proportion of pixels of the corresponding colours (Fig. 4) gives an efficiency of 30%.

Supplementary Table 3: Efficiency of the QR code print. *A power meter was used to collect the light in the central projection (including the zero-order spot) some distance from the print ("power in central projection") and solely within the zero-order spot at a farther distance ("power in zero-order spot"). Further measurements were made, first replacing the print with an unpatterned glass substrate ("transmitted power") and then removing the print altogether ("incident power"). Power is given in units of mW.*

(power in units of mW)	**Blue laser (449 nm)**	**Red laser (638 nm)**
Incident power	1.80	2.10
Transmitted power	1.68	1.90
Power in central projection	0.53	0.64
Power in zero-order spot	0.15	0.34
Glass transmission efficiency	1.68 ÷ 1.80 × 100% = 93%	1.90 ÷ 2.10 × 100% = 90%
Filter transmission efficiency	0.53 ÷ 1.68 × 100% = 32%	0.64 ÷ 1.90 × 100% = 34%
Hologram diffraction efficiency	(0.53−0.15) ÷ 0.53 × 100% = 72%	(0.64−0.34) ÷ 0.64 × 100% = 47%
Overall efficiency of print	(0.53−0.15) ÷ 1.80 × 100% = 21%	(0.64−0.34) ÷ 2.10 × 100% = 14%

Supplementary Note 6 – Pixel arrangements for spatial multiplexing of holograms

Holographic colour prints lie on a continuum between colour images, in which the arrangement of pixels is rigidly defined, and multiplexed holograms, for which the arrangement of pixels is seemingly arbitrary. However, even if the requirement to form a colour image is removed, there are still restrictions on the types of pixel arrangements that can be used for hologram multiplexing. Because our holograms are Fraunhofer holograms that operate in the Fourier domain, the Fourier transform of the (real space) pixel arrangement enters into the determination of the final holographic projections – specifically, *the final holographic projection is the spatial convolution of the designed holographic projection with the Fourier transform of the pixel arrangement*. The implications of this mathematical relationship on the design of multiplexed holograms are elaborated on in the following.

Adopting an idealised matrix representation of the pixel arrangement, the presence or absence of a hologram pixel at each location in space is denoted respectively by an amplitude of one or zero in the corresponding position of a 2D matrix (a binary mask). Then a matrix of ones corresponds to a hologram that completely fills the illuminated area and diverts the entire incident beam to project a desired image, whereas a matrix of zeroes corresponds to an illuminated area unoccupied by hologram pixels such that the incident beam passes straight through and remains as a spot.

In our space-division wavelength-multiplexing scheme, the pixels of each hologram only occupy part of the total area, which gives a "patchy" pixel arrangement on each wavelength channel. When pixels are removed from a complete, unmultiplexed hologram to create a patchy pixel arrangement (introducing zeroes into a matrix of ones), the undiffracted central (zero-order) bright spot increases in intensity at the expense of the projected image. While the projected image might then simply be expected to fade away gradually as pixels are removed, it can in fact become blurred or repeated. This is because the holographic projection is not only affected by the number of pixels remaining, but is also highly sensitive to the locations of the remaining pixels (i.e. the pixel arrangement).

To understand this, consider that the Fourier transform of a constant amplitude profile (the pixel arrangement of a complete, unmultiplexed hologram) is a Dirac delta function and returns an identical projection after convolution. However, the Fourier transform of the pixel arrangement of a patchy hologram is a combination of a Dirac delta and some noise terms which draw power away from the Dirac delta. Convolution with such a "noisy delta" function has the effect of creating unwanted copies of the holographic projection ("ghost images") that weaken and distract from the desired central projection.

These disturbances to the holographic projection can become especially pronounced when the Fourier transform has localised regions of high intensity noise that concentrate the ghost images and make them more apparent – a particularly serious issue when imperfect wavelength selectivity causes them to appear as crosstalk on multiplexed channels. In general, any form of ordering or periodicity in a pixel arrangement will be manifested as clustering or peaks in its Fourier transform and thereby accentuate the crosstalk noise in multiplexed holographic projections, as shown in the simulated far field projections in Supplementary Fig. 10. Compared with a random pixel arrangement which produces three projections with little crosstalk (Supplementary Fig. 10a), a "blocky" pixel arrangement band-limits the Fourier power spectrum, which concentrates ghost images around the central projection and gives it a blurry appearance (Supplementary Fig. 10b). Meanwhile, a periodic pixel arrangement creates regular peaks in the power spectrum, which causes the tiling of ghost images in the Fourier plane (Supplementary Fig. 10c). The crosstalk, which is barely noticeable in Supplementary Fig. 10a, becomes much more apparent in Supplementary Fig. 10b,c as the overlapping of ghost images enhances their visibility not only in their own channels but also on other channels.

Based on the above analysis, the optimal pixel arrangements for multiplexing are those which can spread out the ghost images by diffusing the noise power uniformly across the entire frequency domain to create a flat power spectrum, or equivalently, by generating a white noise signal in real space. We found that a convenient way to achieve a white noise spectrum was to use an error diffusion dithering algorithm to perform the necessary colour matching between the colour image to be printed and the colour palette available.

We used the Floyd-Steinberg error diffusion algorithm implemented in MATLAB as the built-in function **dither**. As compared to naïvely mapping a colour image to a more limited colour palette by directly applying error minimisation to each pixel, dithering diffuses the quantisation error of each pixel over its neighbouring pixels so as to spread out the error uniformly. Doing so improves the appearance of high-error regions with little degradation of low-error regions, thereby increasing the apparent quality of the recoloured image.

Apart from increasing the perceived colour accuracy beyond the results of simple error minimisation algorithms (which improves the quality of the colour print), dithering also minimises occurrence of large single-colour blocks of pixels, typically breaking them up into a complicated halftone pattern of various other colours. This scrambling of the colour pixels helps to randomise the pixel arrangement on each colour channel and generate a flatter power spectrum more similar to that of white noise (which improves the fidelity of holographic projections).

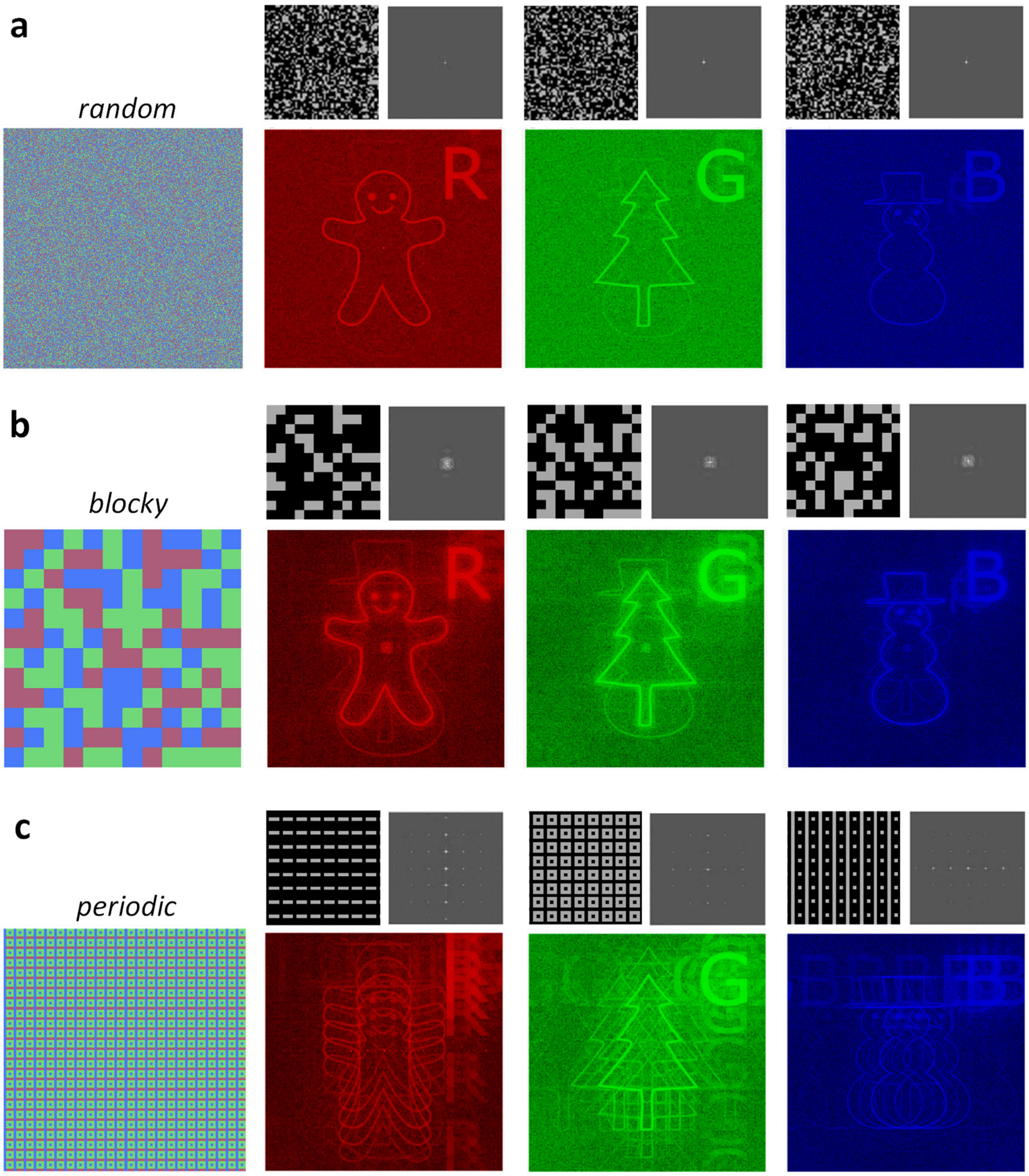

Supplementary Figure 10: Effect of pixel arrangement on multiplexed holograms. *A comparison of 480×480 red, green, and blue (RGB) pixel arrangements for hologram multiplexing and simulated far field holographic projections, based on the RGB laser wavelengths and transmission spectra in Fig. 2. Above each projection is the pixel arrangement for its colour channel, rescaled to show its essential features (left), and the Fourier transform of the pixel arrangement (right).* ***(a)*** *A random pixel arrangement in which individual RGB pixels are interspersed to give a featureless appearance.* ***(b)*** *A "blocky" random pixel arrangement in which randomness is only applied down to a scale of 40-pixel*

blocks. ***(c)*** *A periodic pixel arrangement in which a 4×4 super-pixel is tiled to fill the space. The random arrangement (a) is the most suitable for hologram multiplexing as it accurately reproduces the source images with minimal crosstalk. With the blocky arrangement (b), unwanted "ghost images" are concentrated around the central holographic projection, forming a diffuse glow that highlights the crosstalk in the background. In the periodic arrangement (c), the ghost images are repeated across the Fourier plane at locations determined by the positions of peaks in the Fourier transform of the pixel arrangement.*

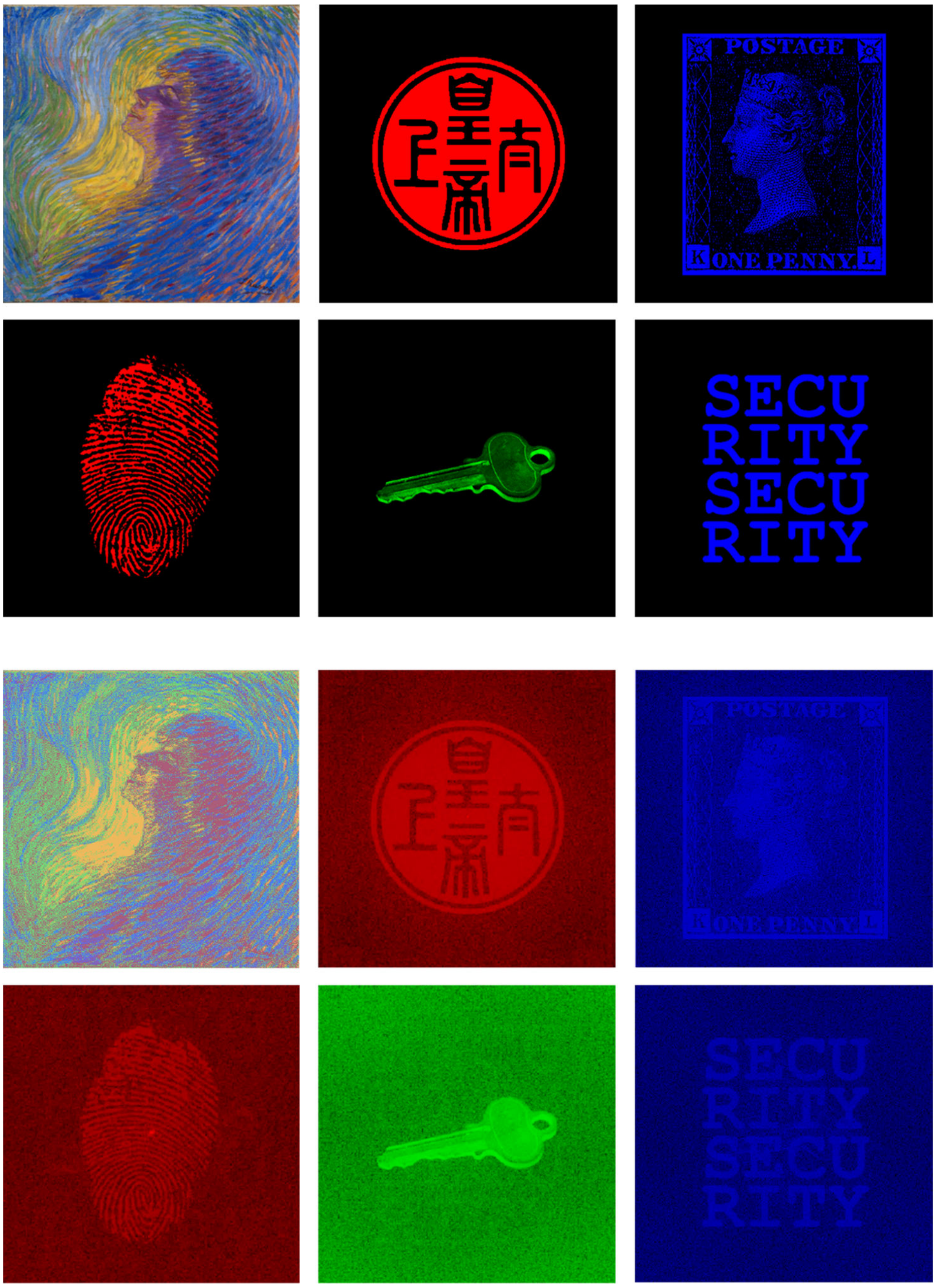

Supplementary Figure 11: Original source images and simulations. *Source images (above) and corresponding simulations (below) of the* Perfume *colour print and holographic projections. Simulations are based on the RGB laser wavelengths and colour filter transmission spectra in Fig. 2 and the pixel layouts from Figs. 3 and 4. The colour print is simulated for a 1931 CIE 2° standard observer viewing under illuminant D65 (white light with a colour temperature of 6500 K).*
